# Latin American Considerations for Infant and Young Child Formulae

**DOI:** 10.3390/nu13113942

**Published:** 2021-11-04

**Authors:** Liliana Ladino, Nathalia Sánchez, Rodrigo Vázquez-Frias, Berthold Koletzko

**Affiliations:** 1 Faculty of Medicine, Research Institute on Nutrition, Genetics and Metabolism IINGM, Universidad El Bosque, Bogotá 110121, Colombia; lladinom@unbosque.edu.co; 2 Nutrition Working Group, Latin-American Society for Pediatric Gastroenterology Hepatology and Nutrition LASPGHAN, Mexico City 11560, Mexico; nutrition@laspghan.org; 3 Research and Education Center on Nutrition, CIENutrition, Bogotá 110221, Colombia; investigacion@cienutrition.org; 4 Department of Gastroenterology and Nutrition, Hospital Infantil de México Federico Gómez, Mexico City 06720, Mexico; 5 Department Pediatrics, Medical Centre of LMU Munich, Ludwig Maximilians University of Munich, Dr. von Hauner Children’s Hospital, 80539 München, Germany

**Keywords:** infant formulae, follow-up formulae, young child formulae, marketing practices, national regulations, influencers

## Abstract

Infant formula is the only acceptable substitute for breastmilk from 0 to 6 months old when human milk cannot be provided in sufficient amounts. Manufacturers have developed options that intend to meet the changing needs of the child aged from six to twelve months (follow-on formulae) and after the age of one year (young child formulae). The international code for marketing breast milk substitute stipulates standards for marketing practices of these products. In Latin America there are local variations of marketing practices. Novel marketing strategies such as advertising through social media and influencers pose new threats for breastfeeding success in Latin America. This review aims to examine variations in local regulations for marketing of infant formulae and to analyze the emerging phenomenon of influencer advertising. We reviewed the local norms for Latin American countries and examined differences and possible gaps. Emerging evidence of influencer marketing was explored. The results indicate that national regulations differ among Latin American countries, particularly with respect to product labelling and the requirement to use a local native language, highlighting the cost of the product, and different regulations prohibiting certain messages and illustrations. Regarding new marketing strategies, there is limited evidence on advertising infant formula through social media influencers, where different categories of marketing strategies can be described. More transparent reporting of social marketing by formula providers and more independent research on novel marketing strategies are needed.

## 1. Introduction

For populations globally, the World Health Organization (WHO) recommends exclusive breastfeeding for about six months with continued breastfeeding along with balanced complementary feeding until at least two years of age, considering the benefits of breastfeeding on risk reduction for infection, physical, mental and social development [1]. For populations with a low burden of infectious disease such as infants in Europe, breastfeeding for at least the first 6 months of life and exclusive breastfeeding for 4-6 months has been recommended [2]. Infants that cannot receive sufficient amounts of human breast milk need safe infant formula of high quality as a breast milk substitute [3].

Formulae for infants are classified into regular formula for generally healthy infants, and formula for special medical purposes for the dietary management of disorders under medical supervision [4]. The regulatory standards of the Codex Alimentarius of the WHO and the Food and Agriculture Organization of the United Nations (FAO) [4], the European Food Safety Authority (EFSA) and the European Commission [5,6] and the United States Food and Drug Administration (FDA) [7] are based on the general agreement that “*Infant formula means a breast-milk substitute specially manufactured to satisfy, by itself, the nutritional requirements of infants during the first months of life up to the introduction of appropriate complementary feeding*”[4].

Since infant formula differs from other foods in that it must satisfy all the nutritional needs of an individual from a single food product, while providing a very high nutrient supply per kg bodyweight during a particularly sensitive and vulnerable period of life, the regulatory standards for infant formulae set much stricter compositional, quality and safety standards than for any other food products [3].

There are also additional ingredients such as nucleotides, docosahexaenoic acid (DHA), arachidonic acid (ARA), oligosaccharides with presumed prebiotic functions, probiotics and complex fat components, which are added to some formulae because they are expected to provide potential benefits on visual and brain functions, the immune and bone system, intestinal microbiota, and other outcomes [8].

Globally and in Latin America, there is a need for enhancing promotion of breastfeeding. Inappropriate marketing practices for infant formulae need to be identified and ended. The international Code for marketing of Breast milk substitutes (BMS) stipulates standards for BMS marketing, which countries have implemented with different variations into national regulations. New challenges arise due to the emergence of social media and influencer marketing of BMS that are not yet fully regulated.

This review aims to examine the characteristics of infant, follow-up and young child formulae, national differences of legal regulations for these products across different Latin American countries and to describe a current marketing strategy for BMS.

## 2. Methods

Within the months of March and June 2021 research of systematic reviews and intervention studies using the term “infant formula”, “infant formula composition”, “formula preparation”, “follow-up formula”, “follow-up formula composition”, “young child formula” and “young child formula composition”, “infant formula marketing”, “formula marketing” and “formula influencers” was carried out in English and Spanish language in the databases: PubMed, Elsevier and Scielo. Similarly, materials related to formula feeding, different types of formula and formula preparation provided by international entities such as WHO, CODEX Alimentarius and UNICEF United Nations children’s Fund were revised.

Additionally, the regulatory norms for BMS marketing for Latin American countries were compiled where available online or through individual experts contacted in the respective countries.

## 3. Infant Formulae

The global standards for infant formula composition were revised by the Codex Alimentarius in 2007 [4] and were informed by recommendations of an international expert group invited by the European Society of Pediatric Gastroenterology, Hepatology and Nutrition (ESPGHAN), published in 2005, which are presented in Table 1.

Infant formula is required to provide a protein content in the range of 1.8–3.0 g/100 kcal (7.3–12% of energy) if based on cows’ or goats’ milk protein [4,6] or a higher protein content if based on soy protein isolate. A minimum content of indispensable amino acids is defined. While cows’ milk usually provides about 70–80% of casein and 20–30% whey proteins, modern formulae are enriched with whey proteins to improve the amino acid supply.

More than a decade ago, the early protein hypothesis was established suggesting that a high protein intake in excess of infant requirements can promote early weight gain (i.e., a rapid increase in infant weight), an increased later obesity risk, along with elevating insulinogenic amino acids, insulin release and insulin-like growth factor 1 (IGF-1) in infant blood and tissues [10]. This hypothesis has been confirmed with the results of the European CHOP (Childhood Obesity Project) randomized trial. Infants were randomized to feeding with a conventional formula with a high protein content, or an isoenergetic formula with a reduced protein content closer to human milk composition [11]. Infant feeding of the higher protein formula resulted in a significantly higher BMI (Body Mass Index) and body fat mass at the ages of 2 and 6 years, and a 2.6 fold higher obesity risk at age 6 years after adjusting for confounders, compared to infant feeding with the lower protein formula [11]. Accordingly, EFSA and the European Commission reduced the range of acceptable protein content in formula for infants, and also the new Codex Alimentarius draft standard for follow-on formula suggests a lower range of acceptable protein content.

Carbohydrates in infant formula usually are in the range of 9–14 g/100 kcal (36–56% of energy) [4,6]. The preferred carbohydrate in infant formulae is lactose, the only digestible carbohydrate in human milk. Lactose has prebiotic effects and benefits for the gut microbiome, intestinal physiology and calcium absorption [12,13].

The fat content and composition in human milk is very variable and is modified by the mother’s diet and other factors [14]. Formula fat composition is very different from human milk fat. Formula fat is usually based on a mix of vegetable oils such as canola, soy, sunflower, palm and coconut oils that are emulsified with soy lecithin. The addition of the long-chain polyunsaturated fatty acids docosahexaenoic acid (DHA) and arachidonic acid (ARA) to infant and follow-on formula is recommended [15]. Table 2 provides a summary of the macronutrient content of infant formulae in 100 mL of a standard dilution and per 100 kcal as stipulated by current regulatory standards.

For the Nutrition Group of the Latin American Society of Pediatric Gastroenterology, Hepatology and Nutrition (LASPGHAN), infant formulae are the only advisable BMS. Infant formula may be fed for the whole first year and beyond.

## 4. Follow-Up Formulae

Even though infant formulae are suitable for the feeding for the duration of the first year of life and beyond, follow-on formulae have been offered since the 1970ies and are now widely used around the world to enable an adaptation of dietary intake to age related changes in needs [16]. According to the Codex Alimentarius, follow-up formula is: “*a food intended for use as a liquid part of the weaning diet for the infant from the 6th month on and for young children*” [17]. In the European food legislation, the compositional requirements for infant and follow-up formula are very similar. The major differences of follow-up formula composition, as compared to infant formula, are an increased iron content (0.5–2.0 vs. 0.3–1.3 mg/100 kcal) and a lower minimum protein content (1.6 vs. 1.8 g/100 kcal), matching the changing requirements over time [6,18]. High iron stores at birth suffice to cover the iron needs for growth during the first postnatal month, compensating for the low iron content of human milk, and they lead to a low postnatal iron absorption. Therefore, a high iron supply with formula in the first 6 months remains mostly unabsorbed and the non-absorbed iron can induce adverse effects on gut microbiota, infection and inflammation, and length growth. Therefore, a low iron supply with formula fed from birth, similar to the low iron supply with breastfeeding, is desirable. However, towards the end of the first year endogenous iron stores become depleted, and an increased dietary iron supply from complementary feed and higher contents in follow-up formula is useful.

Infant protein requirements are related to weight gain velocity which rapidly declines during the first months of life. Accordingly, protein requirements per kg bodyweight at 6 months of age are only 2/3 of those after birth, and protein supply per kg bodyweight in an exclusively breastfed infant at 6 months is only 55% of that after birth [19]. The use of adequately composed follow-up formula can help to better match dietary intake to changing needs with age. LASPGHAN suggests that they can be used for infants older than 6 months without age limit. Lactose should continue to be the main carbohydrate, whereas they should be sucrose free.

Global standards for the follow-up formulae composition were updated by the Codex Alimentarius in 2013 and are currently under review [17]. They were informed by recommendations of international experts coordinated by the Early Nutrition Academy (ENA) [20] (Table 3 provides a summary of the micronutrient content of infant formulae in 100 mL of a standard dilution and per 100 kcal as stipulated by current regulatory standards.

## 5. Young Child Formula

While infant and follow-up formulae serve as a substitute for breast milk, young child formulae used from the age of one year onwards are meant to serve as an alternative to cows’ milk with an improved nutrient composition. Manufacturers have often called such products “*growing up milks*”, a term that is not supported by the pediatric community because it implies that such products would have a functional impact on growth for which there is no evidence whatsoever, therefore the term “*growing up milks*” is considered an unfounded and misleading health claim. The protein content of young child formula is usually lower than that of cows’ milk (CM). Fats are mostly a blend of vegetable oils similar to other regular formulae, and hence they contain more polyunsaturated and less saturated fats than cows’ milk. Many products contain not only lactose but are also sweetened with saccharose (sucrose), which is undesirable. Moreover, the frequent addition of strong flavors and aromas is not supported because it may interfere with the desirable imprinting of taste preferences for normal healthy foods. Given that young children around the world [21] as well as in Europe [22] often have insufficient intakes of critical nutrients such as iodine, iron, vitamin D, alpha-linolenic acid and DHA and too high intakes of animal protein, adequately composed young child formula are one of several options that can contribute to improve dietary quality [21,23]. Recommendations for the composition of young child formula have been published (Table 4).

Across Latin American countries nutritional deficiencies of iron, zinc, vitamin A, vitamin D and other nutrients have been reported with a high prevalence in different Nutrition Surveys of most of the countries in the region. In 2016, UNICEF reported that only 16% of children between 6 and 23 months of age in the world complied with the minimum acceptable diet, defined as those who reach the minimum number of meals plus a minimum number of foods on the previous day [24]. LASPGHAN considers that in the case of Latin America, adequately composed young child formulae replacing part of the dietary CM may have additional benefits for children who have a poor diet [22].

**Figure 1:**
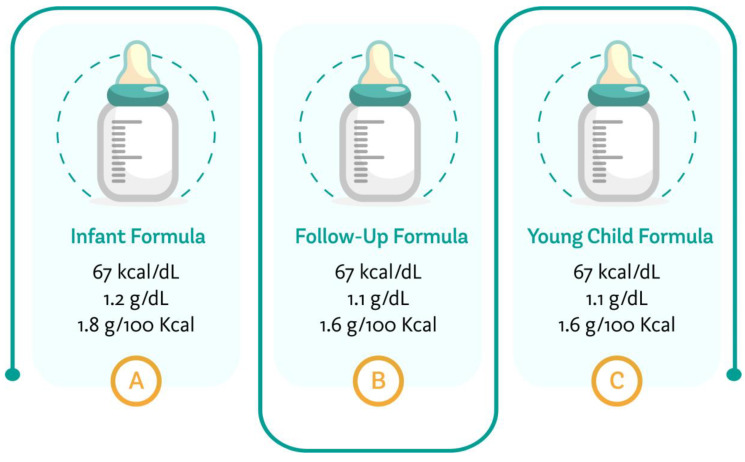
Average energy and minimum protein content of formulae targeting different age groups. Author’s creation, 2021.

Figure 1 summarizes the average energy content of the regular formulae marketed for different age groups, the minimum protein content for infant formulae, follow-up formulae and young child formulae, based on the different requirements available for their composition [4,17,23].

## 6. Presentation, Storage and Preparation of Formulae

Formulae for infants and young children are marketed as powder, ready-to-use formula, or concentrated liquid formula. All have a similar nutritional content, but specifications for preparation, storage and use differ [25].

Powdered formulae should be prepared by diluting the product in warm water. It is essential to use clean potable water, not contaminated with microorganisms, high nitrate contents, or untoward concentrations of trace elements such as fluoride, copper, lead or arsenic. The use of bottled water is recommended when no other source of safe water is available [26]. Families should follow the manufacturer’s specific instructions; they must use the measure or scoop and avoid the use of kitchen utensils or the measure of other products since the correct preparation depends on this [8]. Within the first months of life, it is recommended to provide the formula at a warm temperature. To achieve this, it can be heated in a water bath for 5 to 10 min before offering it. A microwave oven should never be used to heat the formula as this creates the risk of burns caused by “hot spots” in the dilution, which are not detectable when the mother performs the temperature test on her wrist. Regarding storage of formula powder, it is recommended to keep the container in a dark, cool and ventilated place but not in the refrigerator [8]. It is key to instruct parents in proper hygiene practices such as hand washing and the importance of using a clean surface and utensils (containers and bottles) during the preparation and feeding process. It has been found that a large number of parents use improper preparation techniques by not following the manufacturer’s recommendations, such as the order of adding the ingredients. The FAO and WHO recommend that powdered infant formulae should be prepared by first adding the water and then the powder to be able to reconstitute a greater volume than that given merely by water [27]. This extra volume is given by the displacement and hydration of the powder. If not done properly, there can be a change in the energy density of the formula, which may affect the infant’s energy and nutrient intake [28], and it can increase renal solutes load, alter weight gain, cause gastrointestinal discomfort, and even lead to osmotic diarrhea. All of the above, often cause an unnecessary change of the formula, as well as an increase in costs to the health system in the event that this happens at the intra-hospital level or to higher parents and/or caregivers expenses [29].

Table 5 shows the final volume of properly prepared infant formulae.

Prepared formula is a good growth medium for pathogenic microorganisms that may occur in small numbers in the powder and in the surrounding environment when preparing formula, for example Salmonella spp. or Cronobacter spp. If these are sufficiently multiplied in formula after reconstitution they may cause invasive infections, particularly in preterm and term neonates [31]. Therefore, powdered formula should always be freshly prepared for each feed and be fed within a period of 2 h after preparation, while unused prepared formula should be discarded [3]. Hospitals, day care centres and other institutions should follow strict hygienic standards and written guidelines for the preparation and handling of formula. For neonatal wards, the use of liquid formula is encouraged if feasible and affordable.

## 7. Marketing of Formulae for Infants and Young Children

The global paediatric community is a strong supporter of the International Code of Marketing of Breastmilk Substitutes (“*Code of Marketing*”) and its goals to eliminate improper practices of marketing of BMS that may undermine breastfeeding [32,33]. Many paediatric organisations support that no direct marketing to consumers should occur for infant formula and for follow-up formula for older infants, which are generally presented with similar brand names and design as infant formulae, because improper marketing may mislead families to perceive formula feeding as equivalent to breastfeeding and hence may undermine breastfeeding [34]. Similarly, paediatric organisations oppose marketing to consumers for formula for special medical purposes for infants (e.g., special formula for treating cow’s milk allergy, for preterm babies, etc.) because these should only be used upon medical advice and under medical supervision [3]. Therefore, also these products must not be promoted with any type of advertisement directed to parents, such as TV advertisements. Currently, the practice of marketing infant and follow-up formulae in many countries is still less than satisfactory. Paediatricians and their organisations, consumer organisations and governmental bodies should strengthen their joint efforts to end improper marketing practices.

### 7.1. Regulations on Marketing of Infant Formula

**Figure 2:**
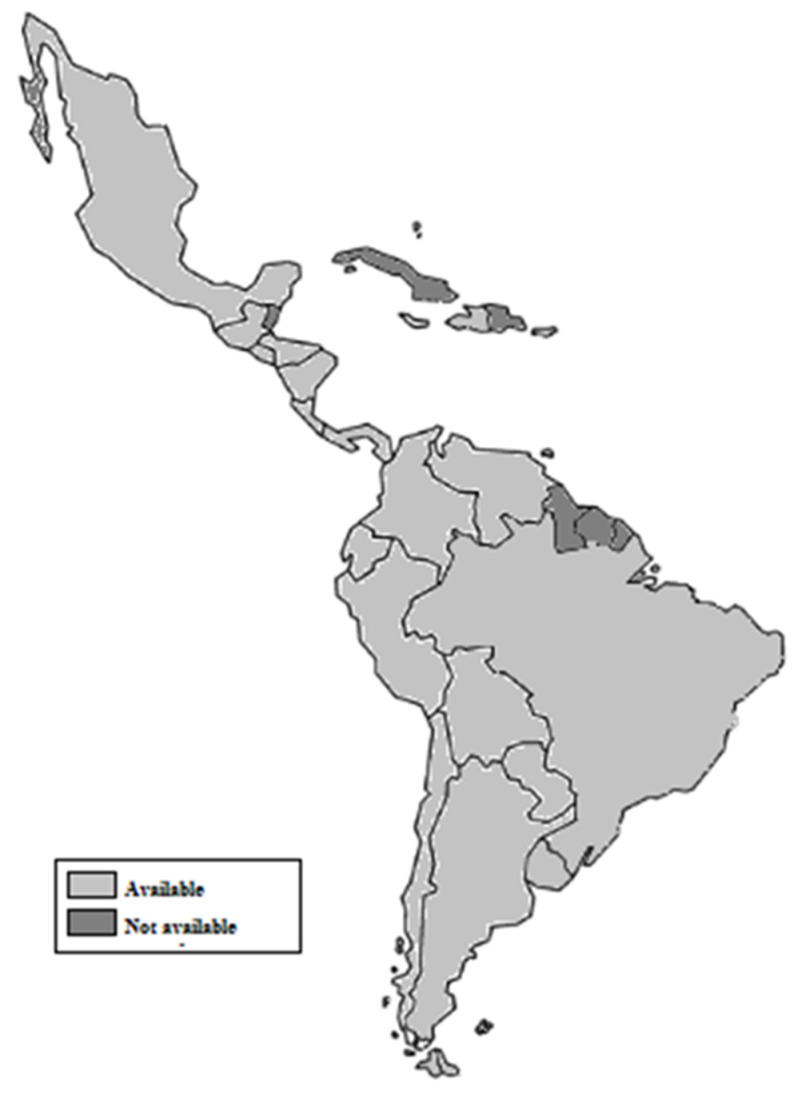
National regulations on infant formula marketing in Latin America available or not available online. Author’s creation, 2021.

The framework for infant formula marketing regulation is the International Code of BMS; however, each country develops their national regulations and can determine standards for industry and the health care sector. The regulations of most countries are available online and were compiled for this review. Figure 2 shows the local current regulation available on line for Latin American countries.

One of the aspects that can be variable is the periodicity for updates. Table 6 and Table 7 provide an overview of the current legal scenario in Latin America and the most recent updates.

Among the divergent aspects in the various national regulations is the different emphasis on the use of local language, the inclusion of labelling aspects such as the cost of using the product for 6 months, the specific prohibition of messages that could lead to doubt of mothers about their capability to breastfeed, and prohibition of some types of illustrations such as animal forms or toys, and other topics such as safe feeding methods.

The existence of different regulations among countries may lead to problems in communication, and the creation of joint policies and protocols for the region, and may facilitate the perpetuation of breaches of the international code for BMS and continuation of inequity in infant health for specific countries.

### 7.2. New Marketing Strategies

With the surge of social media, a new space for novel marketing strategies has emerged. A new phenomenon appeared, the marketing by social media “*influencers*”. An influencer here is defined as a person who is paid by a company to describe and promote its products on social media and to encourage people to buy them [58]. With this and other strategies, the internet is now an important platform to marketing foods including foods for infants, exposing particularly young people that tend to use the internet at a considerable amount of time [59]. This concept is rather new and has not been consistently regulated. Often the payment of an influencer for promoting commercial products or services is not disclosed, and many followers may not be aware of the advertising character of messages that may be presented as personal experience and advice among friends.

BMS regulations in large parts of the world including Latin America prohibit advertisement of standard infant formula to the public. However, the influencer strategy is currently used to promote standard infant formulae and circumvents existing regulations. It is imperative for the International Code of BMS and national regulations to ban this kind of promotion in order to avoid that parents are unduly influenced, and that breastfeeding rates and duration are undermined.

### 7.3. The Role of Influencers

**Figure 3:**
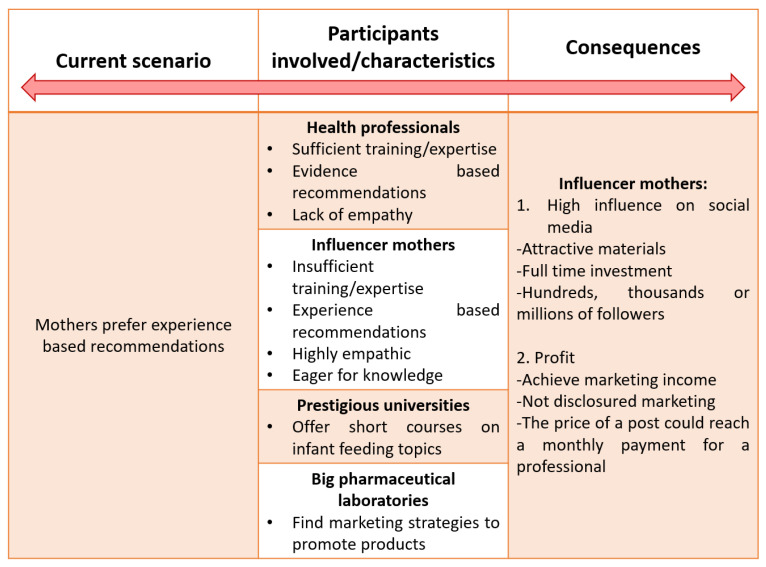
Causes, consequences and actors involved with influencer mothers.

The American Academy of Pediatrics reported that many recommendations followed by mothers regarding infant and young child feeding practices are based on experience and personal recommendations [60] and not evidence-based [61] (Figure 3). Qualitative studies show that mothers more often are guided by recommendations from another mother rather than a health professional [62], influenced by an often-perceived lack of empathy from health professionals. This space has also been increasingly filled by different types of “*influencers*” using social media. More and more, marketing messages are shared by social media influencers worldwide, with little information available in the scientific literature addressing it. Here we describe the authors’ observations in social media in Latin America (specially Colombia and Mexico) and Europe (specially Germany). The first type of influencer is highly related to the perception of mothers of a lack of empathy from the health professionals. Influencers obtain information on infant feeding from various credible sources any may even use certification e.g., from Massive Online Open Courses (MOOC). Claiming the authority gained from this training, some produce educational material in different formats or styles (posts, short videos, live streaming, etc.) and share it on social media providing recommendations on different aspects of breastfeeding, bottle and complementary feeding that should only be provided by a health professional, and even promote specific products.

A second type of influencer is equally connected to the preference of mothers to take advice from another mother. Particularly effective are models that are providing these recommendations and are popular as actresses, singers or celebrities in general. Mothers with this profile can narrate their experience with pregnancy and feeding process for their infants on social media.

These messages are frequently indirectly linked to positive characteristics or emotions such as “*empowerment*“, which can be equivocal in this situation, especially if this represents a profit for the mother and companies find a place to promote products like formulae without even a disclosure of the paid publicity.

A third problematic scenario in this regard is the use of social media by health professionals. Using equivocal denominations for short trainings that could undermine the postgraduate degrees and lead to misinformation by the followers. Similarly, the production of recommendations that are not completely linked to their area of expertise.

## 8. Feeding and Infant Health

Exclusive feeding with human milk is consider the ideal feeding method during the first six months of life. Subsequently, adequate complementary feeding is of great importance since part of the calories and nutrient requirements are not met by human milk alone from the age of about 6 months onwards [62].

While during the first six months of age children’s’ health depends completely on the mother’s diet, after the introduction of different foods health will also depend on the foods that are provided to the child. An appropriate nutrition during the first 2 years of age and within the preschooler years (until 5 years of age) is fundamental due to its impact in different aspects of health and development [63]. The benefits of breastfeeding in the short term include a reduction in infectious disease and children mortality and in the long-term cognitive development the prevention of chronic health conditions such as obesity and diabetes in the future [63]. Complementary feeding and nutrition of the young child and preschooler years is also crucial due to the establishment of eating habits which are directly related to microbiota homeostasis which is accomplished by the age of three [64,65,66].

It has been described how the first 1000 days of life are determining for children Health and programming future health in the adult life. A period of even 2000 days has been proposed as being important for the establishment of gut microbiota. Table 8 describes the distribution of these first 2000 days of life.

However, in Latin America often time breastfeeding and complementary feeding recommendations are not met. In combination with other circumstances (often linked to poverty) such as the absence of clean potable water, inadequate hygienic or sanitation conditions, wrong preparation of infant formula, and other risk factors, considerable rates of preventable infant and young child morbidity and mortality due to infectious respiratory and gastrointestinal disease persist. A complex interaction results since malnutrition makes children more susceptible to infection, and infections lead to aggravate malnutrition by decreasing appetite, inducing catabolism, and increasing demand for nutrients [68].

According to UNICEF, diarrhea is a leading death cause of young children, accounting for approximately 8% of all deaths among children under age 5 worldwide in 2017. This translates to over 1400 young children dying each day, or about 525,000 children a year [69]. Pneumonia kills more children than any other infectious disease, claiming the lives of over 800,000 children under five every year, or around 2200 every day. Globally, there are over 1400 cases of pneumonia per 100,000 children, or 1 case per 71 children every year [70]. Recommended preventive strategies for both these conditions include the promotion of adequate nutrition for mothers and children, breastfeeding promotion and support, vaccination, micronutrient supplementation (such as zinc, vitamin A), hand washing with soap, and the prevention and treatment of co-morbidities (such as HIV) [71].

## 9. Conclusions

Breastfeeding is the ideal feeding method for infants. BMS supplement or replace human milk when it is available in sufficient amounts, but do not provide the same benefits of breastfeeding such as prevention of infectious diseases, promotion of cognitive development, mother infant bonding, and risk reduction of maternal diseases. Follow-up formulae are marketed for the changing needs of the infant. Young child formulae can have a special advantage when used for children with a poor dietary quality. For all these products a correct preparation and hygienic conditions are of key importance. The regulation for marketing of infant formulae varies among different countries in Latin America. Among the aspects that differ are the specification for the use of local language, and the degree of prohibition of specific messaging or illustrations. Social media influencers are an increasingly important source of information on infant feeding decision for families, and they often implement marketing of commercial products against payment, which often is not transparently disclosed, circumventing established restrictions on the marketing of BMS. Three types of influencers that induce risk include mothers who took short trainings on infant feeding, the use of celebrities to promote products or feeding practices, and health professionals who use equivocal denomination for their professional training on social media. Responsibility for these worrying developments lies not only with individual influencers, but to a large extent with the commercial powers who provide financial incentives to them for marketing products. There is an urgent need for stakeholders such as governmental bodies, health care professionals and their associations, and parent and consumer organizations to jointly put pressure on infant food manufacturers to end such inappropriate marketing practices.

## Figures and Tables

**Table 1:** Recommendation on the composition of infant formulae by an international expert group coordinated by the ESPGHAN [9].

Component/Unit	Mínimum	Máximum
Energy kcal/100 mL	60	70
Proteins g/100 kcal		
Cow’s milk protein	1.8	3
Soy protein isolates	2.25	3
Hydrolyzed cow’s milk protein	1.8	3
Fats		
Total fat, g/100 kcal	4.4	6
Linoleic acid, g/100 kcal	0.3	1.2
α-Linolenic acid, mg/100 kcal	50	n.s.
Ratio linoleic/α-Linolenic acids	5:1	15:1
Lauric + myristic acids, % of fat	n.s.	20
Trans fatty acids, % of fat	n.s.	3
Erucic acids, % of fat	n.s.	1
Carbohydrates		
Total carbohydrates, g/100 kcal	9	14
Vitamins		
Vitamin A, µg RE/100 kcal	60	180
Vitamin D3, µg/100 kcal	1	2.5
Vitamin E, mg α-TE/100 kcal	0.5	5
Vitamin K, µg/100 kcal	4	25
Thiamin, µg/100 kcal	60	300
Riboflavin, µg/100 kcal	80	400
Niacin, µg/100 kcal	300	1,500
Vitamin B6, µg/100 kcal	35	175
Vitamin B12, µg/100 kcal	0.1	0.5
Pantothenic acid, µg/100 kcal	400	2,000
Folic acid, µg/100 kcal	10	50
Vitamin C, mg/100 kcal	10	30
Biotin, µg/100 kcal	1.5	7.5
Mineral trace elements		
Iron (formula based on cow’s milk protein and protein hydrolysate mg/100 kcal)	0.3	1.3
Iron (formula based on soy protein isolate) mg/100 kcal	0.45	2
Calcium mg/100 kcal	50	140
Phosphorus (formula based on cow’s milk protein and protein hydrolysate, mg/100 kcal)	25	90
Phosphorus (formula based on soy protein isolate) mg/100 kcal	30	100
Ratio calcium/phosphorus mg/mg	1:1	2:1
Magnesium mg/100 kcal	5	15
Sodium mg/100 kcal	20	60
Chloride mg/100 kcal	50	160
Potassium mg/100 kcal	60	160
Manganese µg/100 kcal	1	50
Fluoride µg/100 kcal	n.s.	60
Iodine µg/100 kcal	10	50
Selenium µg/100 kcal	1	9
Copper µg/100 kcal	35	80
Zinc mg/100 kcal	0.5	1.5
Other substances		
Choline mg/100 kcal	7	50
Myo-inositol mg/100 kcal	4	40
L-Carnitine mg/100 kcal	1.2	n.s.

**Table 2:** Compositional requirements for infant formula based on cows’ milk proteins.

	100 mL	100 Kcal
Kcal	60–70	100
Protein g	1.1–2.1	1.8–3.0
Fat g	2.6–4.2	4.4–6.0
Carbohydrates g	5.4–9.8	9.0–14.0

**Table 3:** Recommended composition of follow-up formula for older infants by an international expert group coordinated by the Early Nutrition Academy [20].

Component/Unit	Minimum	Maximum	Guidance Upper Level
Energy kcal/100 mL	60	70	
Proteins			
Cow’s milk protein g/100 kcal	1.7	2.5	
Soy protein isolates g/100 kcal	2.1	2.5	
Fats			
Total fat, g/100 kcal	4.4	6.0	
Linoleic acid, g/100 kcal	0.3		1.4
α-Linolenic acid, mg/100 kcal	50	n.s.	
Ratio linoleic/α-Linolenic acids	5:1	15:1	
Lauric + myristic acids, % of fat	n.s.	20	
Trans fatty acids, % of fat	n.s.	3	
Erucic acids, % of fat	n.s.	1	
Phospholipids mg/100 kcal			550
Carbohydrates			
Total carbohydrates	9	14	
Vitamins			
Vitamin A, µg RE/100 kcal	60	180	
Vitamin D3, µg/100 kcal	1	4.5	
Vitamin E, mg α-TE/100 kcal	0.5		5
Vitamin K, µg/100 kcal	4		27
Thiamin, µg/100 kcal	60		300
Riboflavin, µg/100 kcal	80		500
Niacin, µg/100 kcal	300		1,500
Vitamin B6, µg/100 kcal	35		175
Vitamin B12, µg/100 kcal	0.1		1.5
Folic acid, µg/100 kcal	10		50
Pantothenic acid, µg/100 kcal	400		2000
Vitamin C, mg/100 kcal	10		70
Biotin, µg/100 kcal	1.5		10
Minerals and trace elements			
Iron (formula based on cow’s milk protein), mg/100 kcal	1.1	1.9	
Iron (formula based on soy protein isolates), mg/100 kcal	1.3	2.5	
Calcium, mg/100 kcal	50		180
Phosphorus, mg/100 kcal	25		n.s.
Magnesium, mg/100 kcal	5		15
Sodium, mg/100 kcal	20		60
Chloride, mg/100 kcal	50		160
Potassium, mg/100 kcal	60		180
Manganese, µg/100 kcal			100
Iodine, µg/100 kcal	10		60
Selenium, µg/100 kcal	1		9
Copper, µg/100 kcal	35		250
Zinc, mg/100 kcal	0.5		1.5
Other substances			
Choline, mg/100 kcal	7		150
L-Carnitine, mg/100 kcal	1.2	n.s.	
Taurine, mg/100 kcal	n.s.	12	
Total added nucleotides, mg/100 kcal	0		10.8

**Table 4:** Compositional requirements of young child formula for young children aged 12–36 months proposed by an International Expert Group co-ordinated by the Nutrition Association of Thailand and the Early Nutrition Academy. Modified from [21].

Component	Minimum	Maximum	Guidance Upper Level
Energy, kcal/100 mL	45	70	
Proteins g/100 kcal			
Cow’s milk protein, g/100 kcal	1.6	2.7	
Soy protein isolate, g/100 kcal	2	2.7	
Fats			
Total fat. g/100 kcal	4.4	6.0	
Linoleic acid, mg/100 kcal	500	NS	
Alpha-linolenic acid, mg/100 kcal	50	NS	
*Trans* fatty acids, % of fat	NS	2	
DHA ^1^, % of fat	0.3	NS	
Carbohydrates			
Total carbohydrates, g/100 kcal	9	14	
Vitamins			
Vitamin A ^2^, mcg RE/100 kcal	60		180
Vitamin D, mcg/100 kcal	1.5		4.5
Vitamin B12, mcg/100 kcal	0.15		0.75
Folic acid, mcg/100 kcal	20		100
Vitamin C, mg/100 kcal	4.5		22.5
Minerals and trace elements			
Iron ^3^ (formula based on cow’s milk protein), mg/100 kcal	1		3
Iron (formula based on soy protein isolates), mg/100 kcal	NS		NS
Calcium, mg/100 kcal	200		NS
Sodium, mg/100 kcal	25		75
Iodine, mcg/100 kcal	12		36
Zinc, mg/100 kcal	0.6		1.8

NS, Not specified; DHA, Docosahexaenoic acid. ^1^ The addition of DHA is optional. ^2^ 1 mcg RE (retinol equivalent) = 1 mcg all-trans retinol = 3.33 IU vitamin A. Retinol contents shall be provided by preformed retinol, while any contents of carotenoids should not be included in the calculation and declaration of vitamin A activity. ^3^ The bioavailability is approximately 10%.

**Table 5:** Concentration and Final Volume of Properly Prepared Formulae. Modified from [30].

Water (mL/ounces)	Powder (g)	Displaced Water (mL *)	Final Volume (mL)	Powder Energy (kcal)
30/1	4.3	3.3	33	22
60/2	8.6	6.6	66	44
90/3	12.9	9.9	99	66

* Considering that 1 g of powder displaces 0.77 mL and that they are concentrated at 13%, so they provide 0.67 kcal/mL (≈20 kcal/ounce).

**Table 6:** Regulations on BMS in Latin American countries.

Country	Year	Name and Number	Institution
Ecuador [35]	1995	Law 101, Official Register No. 814	Health Ministry
Perú [36]	2006	Supreme Decrete N 009	Health Ministry
Colombia [37]	1992	Decrete 1397	Health Ministry
Nicaragua [38]	1999	Law 295	Health Ministry
Panamá [39,40]	1995 2012	Law 50 Decrete 1457	Health Ministry
Estados de Unidos de México [41,42]	2012 2018	Norm131 Bill 050	Executive power Health Secretary
República Dominicana [43,44]	1996 1995	Law No 8 Decrete 31	Congress of the republic Congress of the republic
Argentina [45,46]	2013 1969	Law 26.873 National Law 18284	Congress Public Health Secretary
Chile [47,48]	1996 2019	Decrete 977 Law 21155	Health Ministry Ministry of woman and gender equity
Bolivia [49]	2006	Law 3460	Congress
Guatemala [50]	1983	Law 66–83	Health and social assistance Ministry
El Salvador [51]	2013	Decrete 404	Legislative branch
Paraguay [52]	1999	Law 1.478	Congress
Costa Rica [53]	1995	Law 7430	Health Ministry
Honduras [54]	2013	Decrete 231	Congress
Brazil [55]	2018	Decrete Nº 9.579	Executive branch
Venezuela [56]	2007	Law for protection, promotion and support of breastfeeding	National assembly Permanent commision for family, woman and youth
Uruguay [57]	2017	File 2062	House of representatives

**Table 7:** Items related to BMS regulated in different Latin American countries.

Country	Label: State Human Milk Is Superior and the Ideal Feeding Option	Label: State Proper Manipulation, Storage and Preparation	Label: State Risk of Inappropriate Preparation or Non-Hygienic Conditions	Label: Pictures or Graph That Suggest Formula Is an Ideal Feeding Method	Label: Forbids the Use of the Terms Humanized or Maternalized Milks	Label: Forbids the Use of Pictures of Children, Feeding Bottles, Toys or Animals (Except for Preparation Directions)	Label: Official Language and Native Language Adaptations	Label: Logo or Brand Name <20% of Space in Label	Label: It Should Only Be Used under Medical Prescription	Approximate Cost for Using the Product for 6 Months	Label: Risks for Using Feeding Bottles	Label: Describing Best Way to Feed (Cup, Spoon)
Ecuador [35]	X	X	X	X	X	X	X	---	---	---	---	---
Perú [36]	X	X	---	X	X	X	X	---	---	---	---	---
Colombia [37]	X	X	X	X	X	X	---	---	---	---	---	X
Nicaragua [38]	X	X	X	X	X	X	X	---	---	---	---	---
Panamá [39,40]	X	X		---	X	X	X	X	X	---	---	---
Estados de Unidos de México [41,42]	X	X	X	X	X	---	---	---	X	---	---	---
República Dominicana [43,44]	X	X	X	X	X	X	X	---	X	X	X	---
Argentina [45,46]	X	X	X	X	X	X	X	---	X	---	---	---
Chile [47,48]	X	X	---	X	X	X	----	----	X	---	---	---
Bolivia [49]	X	X	---	X	X	X	X	---	---	X	---	---
Guatemala [50]	X	X	---	X	X	X	---	---	---	---	---	---
El Salvador [51]	---	---	---	X	X	X	X	---	---	---	---	---
Paraguay [52]	X	X	Risk of feeding wrong age group	---	X	---	X	---	---	---	---	---
Costa Rica [53]	X	X	X	X	X	X	X	---	X	---	---	---
Honduras [54]	X	X	X	X	X	X	---	---	---	---	---	---
Brazil [55]	X	X	X	X	X	X	---	---	X	---	---	---
Venezuela [56]	X	X	X	X	X	X	X	---	X	---	---	X
Uruguay [57]	X	X	X	X	X	X	X	---	X	X	X	---

The X in the table means this aspect is included in the local norm for BMS regulation.

**Table 8:** Nutrition during the First 2000 days distribution.

Pre-pregnancy	500 days
Pregnancy	270 days
First two years of age	730 days
Preschooler years	500 days

Based on Ladino L, 2018 [67].
